# An exploratory comparison of region-specific feather microstructure in two Eurasian tree sparrow populations across altitudes

**DOI:** 10.7717/peerj.21803

**Published:** 2026-10-22

**Authors:** Fangyuan Liu, Tonghe Zhang, Xu Liu, Qian Zhang, Tiantian Zhang, Juyong Li, Minghui Zhao, Guanqun Kou, Limin Wang, Yang Wang, Dongming Li

**Affiliations:** 1 Hebei Key Laboratory of Animal Physiology, Biochemistry and Molecular Biology, College of Life Sciences, Hebei Normal University, Shijiazhuang, China; 2 Hebei Collaborative Innovation Center for Eco-Environment, Hebei Normal University, Shijiazhuang, China; 3 Cangzhou Normal University College of Life Science, Cangzhou, China

**Keywords:** Eurasian tree sparrow, Feather barbule, High-altitude, Thermal stability, Sex differences, Region-specific

## Abstract

High-altitude environments impose simultaneous challenges to avian flight and thermoregulation, potentially shaping variation in feather microstructure. We conducted an exploratory comparison of feather microstructural traits across feather regions, sexes, and altitudinal populations in the Eurasian tree sparrow (*Passer montanus*). Using scanning electron microscopy, we quantified vane- and downy- feather traits, including barbule length and density, hamuli number, cilia number, ventral teeth number, and internode length and width. We further complemented these analyses with thermogravimetric and calorimetric measurements of breast feathers. Our results showed that feather microstructure was strongly region-specific: tail and wing feathers consistently exhibited interlocking structures (similar hamuli, cilia, and ventral teeth), whereas back and breast feathers showed nodal features. In contrast, population- and sex-associated variation appeared limited and localized to body-feather nodal architecture: high-altitude (HA) females had longer breast internodes than low-altitude (LA) females, and breast internodes were narrower than back internodes in LA birds but not in HA birds. Thermogravimetric and calorimetric analyses revealed similar breast-feather decomposition profiles between populations, consistent with no major difference in keratin thermal decomposition. Our results suggest that feather microstructure in this widespread species is primarily dominated by feather-region specialization, with preliminary evidence of localized population- and sex-associated variation.

## Introduction

Avian feathers are among the most emblematic and functionally innovative epidermal structures in vertebrates. Built primarily from β-keratin-based composite materials, feathers assemble into hierarchical rachis-barb-barbule networks whose multiscale organization (*e.g.*, barbs, barbules, hooklets, and medullary/cortical structure) enables low mass, high damage tolerance, and functional versatility spanning insulation, waterproofing, signaling, and flight (Stettenheim, 2000; Prum & Williamson, 2001; Gill, 2007). Because feather performance emerges jointly from morphology (micro-/nano-architecture) and composition (keratin chemistry, pigments, and associated compounds), selection can act on multiple levels, from barbule interlocking and branching density to material stability and wear resistance, often under competing functional demands (Bonser, 1995; Doucet et al., 2006; Fraser & Parry, 2011). Yet, despite extensive interspecific descriptions of plumage diversity, we still lack a clear understanding of how fine-scale feather microstructure varies within species across environmental gradients and between sexes, and how such variation maps onto functionally distinct feather regions, an essential step for linking macroscopic phenotype, microstructure, and fitness (Pigliucci, 2003; Piersma & Van Gils, 2011).

From developmental and evolutionary perspectives, feathers can be interpreted as products of conserved integumentary organizational principles, in which morphological diversity emerges from modular developmental units operating under spatially restricted patterning systems (Chen et al., 2021; Dhouailly, 2023; Chuong et al., 2025). Variation in feather microstructure is therefore likely shaped by hierarchical developmental processes associated with follicular patterning and tissue differentiation, rather than representing purely independent morphological variation. Feather morphology also reflects a deep evolutionary history shaped by stepwise modifications of ancestral epidermal structures, rather than linear adaptive optimization (Xu et al., 2014; Benton et al., 2019; Xu & Barrett, 2025). Under this perspective, present-day feather structures represent a mosaic of evolutionary modifications that may retain traces of ancestral architectural constraints. Molecular evidence further indicates that regulatory gene networks and genomic patterning systems contribute to the spatial organization of feather traits (Chen et al., 2015; Boer, Van Hollebeke & Shapiro, 2017; Ng & Li, 2018). Together, these processes provide a mechanistic basis for understanding how feather microstructure can be both developmentally constrained and regionally differentiated across the body.

Pennaceous (contour-type) feathers constitute the dominant and structurally elaborate feather type across the body surface in most birds. They comprise a central rachis and two coherent vanes formed by closely packed barbs and barbules; barbules often bear hooklets (hamuli) that engage adjacent barbules to create a “zipper-like” interlocking system, maintaining vane integrity and producing a continuous aerodynamic and water-shedding surface (Stettenheim, 2000; Gill, 2007). Beyond this general blueprint, a hierarchy of microstructural elements, including barbs, barbules, hooklets, cilia, ventral teeth, and internodal patterning in downy portions, contributes to biomechanical and thermoregulatory roles. For example, barbule length and density influence vane stiffness and aerodynamic function, whereas cilia and ventral teeth help govern the integrity of the interlocking system, especially in flight feathers (Kovalev, Filippov & Gorb, 2014; Sullivan et al., 2016; Matloff et al., 2020). In downy regions, internode dimensions and spacing affect structural porosity and heat retention. While these traits vary widely among taxa (Lei et al., 2002; Stuart-Fox et al., 2018; Kashyap et al., 2024), the extent to which they diverge within a species, especially across multiple feather regions and between sexes, remains undercharacterized (D’Alba et al., 2017; De Zwaan, Greenwood & Martin, 2017; Barve & Cadena, 2022).

Functional specialization across feather tracts is shaped not only by biomechanics but also by conserved developmental and evolutionary constraints, with different feather types exhibiting distinct degrees of morphological plasticity (Chen et al., 2021; Terrill & Shultz, 2023; Liu et al., 2026). Feathers are also functionally modular across the body, with different tracts facing distinct mechanical and ecological demands. Back and breast feathers contribute disproportionately to insulation and protection, whereas wing remiges and tail rectrices form aerodynamic surfaces that directly determine lift, thrust, stability, and manoeuvrability (Bonser, 1995; Videler, 2005). These differences imply unequal evolutionary constraints: interlocking and vane-maintenance structures in tail and wing feathers may be more conserved because small changes can compromise flight performance, whereas body feathers may accommodate more variations in traits related to insulation and boundary-layer properties (Bonser, 1995; Stettenheim, 2000; Videler, 2005). Therefore, simultaneous comparisons among back, breast, tail, and wing feathers are necessary to distinguish broadly conserved microstructural modules from those that are more labile and potentially responsive to local environments.

High-altitude environments sharpen these questions by imposing strong yet potentially conflicting demands on plumage. Birds at high altitudes encounter low temperatures and reduced air density, conditions that can simultaneously challenge thermoregulation and flight performance (Altshuler & Dudley, 2006; Scott, 2011; Kiat, Vortman & Sapir, 2019; Martin et al., 2023). Cold conditions increase the value of insulation, while low-density air can alter aerodynamic requirements, potentially changing selection on tail and wing feathers that primarily serve flight (Hendrickx-Rodriguez & Lentink, 2025), relative to body feathers that contribute more directly to heat retention (Pap et al., 2020). Sex adds a further axis of variation: even in species with limited visible sexual dichromatism, males and females can differ in body size, activity, reproductive allocation, and exposure to abrasion or parasites, which can generate sex-dependent microstructure or sex-by-environment interactions (Badyaev & Hill, 2003; Pigliucci, 2003; Gao et al., 2025). At the same time, strong functional constraints could limit divergence in certain microstructures (*e.g.*, components critical for vane interlocking; Barve et al., 2021), making it important to evaluate which traits are flexible *versus* conserved.

Critically, microstructural measurements alone cannot determine whether population differences reflect changes in microstructural architecture, material composition, or both. Thermal-decomposition (*e.g.*, TGA/DTG) profiles provide an independent and complementary line of evidence of feather material properties by characterizing mass-loss stages and thermal stability associated with keratin and associated components; such approaches are widely used for keratin fibres and feather-derived keratin to infer compositional and stability differences (Feughelman, 2002; Fraser & Parry, 2011; Qin et al., 2023). Integrating microstructure with thermal decomposition can therefore test whether any observed phenotypic divergence has a detectable material-property signature, while also allowing explicit support for similarity (*i.e.,* comparable thermal profiles) when material composition appears invariant.

The Qinghai-Tibet Plateau (QTP), the world’s largest and highest plateau, provides an extreme setting to test these ideas, combining chronic cold, hypoxia, low air density, and strong winds that can affect both feather function and degradation processes (Sun et al., 2016; Sun et al., 2023; Sun et al., 2026). The Eurasian tree sparrow (*Passer montanus*, ETS), a widespread human commensal passerine, occupies a broad elevational range from lowlands to over 4,500 m on the QTP and is inferred to have colonized the plateau in association with human agriculture in the recent past (∼2,600 years; Qu et al., 2020). The ETSs on QTP show divergence in morphology and multiple physiological traits relative to their lowland counterparts (Ding et al., 2021; Nabi et al., 2021; Li et al., 2021; Kou et al., 2022; Sun et al., 2023), making them a promising model for investigating the extent of tract-specific and sex-specific variation in relation to altitude.

Here, we compare males and females from high-altitude (HA) and low-altitude (LA) ETS populations and quantify microstructure in four feather regions (back, breast, tail, and wing). We measure traits linked to vane integrity and function (barbule length and density, hamuli number, cilia and ventral teeth numbers) and insulation-related traits in downy/nodal regions (internode dimensions). In parallel, we use thermal-decomposition profiles to test whether population differences in breast feathers, where thermoregulatory selection is expected to be most direct, are accompanied by detectable shifts in keratin decomposition profiles. Given the functional constraints on plumage, we explored the possibility that altitude-related variation—if present—will be region-specific and potentially sex-dependent, and that somatic microstructural modules (particularly those central to aerodynamic integrity) may remain conserved under strong functional constraints. This study is intended as an exploratory assessment of within-species feather microstructural variation across regions and environmental contexts, rather than a definitive test of population- or sex-level divergence.

## Materials and Methods

### Study sites, capture, and sampling

Wild adult ETSs were captured in January 2019 using mist nets at (i) a high-altitude site on the Qinghai-Tibet Plateau (HA: 3,230 m in Jiangxigou Town, Qinghai Province, 36.62°N, 100.28°E) and (ii) a low-altitude site in the suburban area of Shijiazhuang, Hebei Province, China (LA: 80 m; 38.26°N, 114.19°E). Based on NASA POWER climate data (2020–2025), the high-altitude site is characterized by a cold alpine climate with a mean annual temperature of ∼2 °C, mean relative humidity of ∼55%, and an average wind speed of ∼2.7 m s^−1^. The low-altitude site exhibits a temperate continental climate with a mean annual temperature of ∼14 °C, mean relative humidity of ∼58%, and an average wind speed of ∼1.9 m s^−1^. From each bird, we collected four to five feathers per region from the tail (rectrices), secondary flight feathers (remiges), back, and breast feathers. Feathers were stored dry in clean, labeled envelopes until processing. The ETS is largely sexually monomorphic in external appearance, making reliable sexing in the field difficult. Therefore, we used molecular sex identification. Briefly, after capture, we collected ∼80 µL of blood from the brachial (subwing) vein using a sterile needle, stored it in heparinized microhematocrit capillary tubes, and kept the samples at −80 °C until DNA extraction. Genomic DNA was then extracted using a blood genomic DNA small-volume extraction kit (CWBIO, Cat. CW2087; Li et al., 2021), and sex was determined by PCR amplification of the CHD1 gene to distinguish males from females (Round et al., 2007). Finally, we sampled four individuals per population (two males and two females). All animal procedures were conducted in strict accordance with institutional and national guidelines for the care and use of laboratory animals. The study was reviewed and approved by the Biomedical Ethics Committee of Hebei Normal University (Protocol Number: 2019SC10). The approved protocol included the capture of wild individuals, blood sampling, and subsequent experimental procedures. All efforts were made to minimize the number of animals used and to reduce potential pain and distress during handling and sampling.

### Scanning electron microscopy

To remove surface lipids and contaminants, feathers were defatted in 95% ethanol: ether (1:1, v/v) for 15 min, rinsed in absolute ethanol for 3 min, and air-dried at room temperature. Dried feathers were mounted on aluminum stubs using double-sided carbon tape. Because feathers are rigid, critical-point drying was not required. The mounted samples were sputter-coated with gold for 2–3 min under vacuum for conductivity. Feathers were examined using a scanning electron microscope (HITACHI S-570, Tokyo, Japan) at an accelerating voltage of 15 kV. For each feather region per individual, 3–4 fields of view were imaged, with each field of view derived from a single feather sample (yielding 9–12 micrographs per region when multiple feathers were imaged). Imaging targeted vane areas where barbules and interlocking structures were clearly visible. Tail and wing feathers contained hooked barbules bearing hamuli and paired cilia, and unhooked barbules with ventral teeth; back and breast feathers additionally contained nodal barbules with periodic nodes and spinous processes. For back and breast feathers, measurements of barbule length and density were taken from the distal pennaceous portion of the vane, whereas internode-related measurements were restricted to the plumulaceous (downy) regions. SEM images were used to confirm feather-region-specific barbule types and to quantify traits.

We quantified the following traits in all four regions (back, breast, tail, and wing): (i) barbule length (µm): measured along the barbule from its base at the barb to its distal end; (ii) barbule density (no. per µm): counted as the number of barbules per mm along a standardized segment of a barb; (iii) hamuli count (no.): counted on hooked barbules within a standardized image window; when present at lower density (typically in back/breast feathers), hamuli were still scored using the same counting rule to enable among-region comparisons. We quantified cilia number and ventral teeth number only in tail and wing feathers: (i) cilia count (pairs): cilia occur as bilateral pairs; therefore, we recorded the number of cilia pairs per barbule; (ii) ventral teeth count (no.): counted on unhooked barbules. We quantified internode length and width only in back and breast feathers (downy/nodal regions): in nodal barbules with periodic nodes, internode length was measured between adjacent nodes; internode width was measured at the internode midpoint.

### Thermal analyses

Among the four regions, thermal analyses between altitudes were performed on breast feathers, as breast feathers are most directly associated with thermoregulatory function. Breast feathers were collected from adult ETS sparrows from HA and LA populations (*n* = 4 each). Feathers were flash-frozen in liquid nitrogen and ground to a fine powder with a pre-chilled mortar and pestle. Thermal decomposition was quantified using a simultaneous thermal analyzer (PerkinElmer STA800; Waltham, MA, USA) equipped with DSC8500 and TGA8000 modules. Measurements were conducted under nitrogen (≥99.99% purity), heated from room temperature to 800 °C at 10 °C/min. TG, derivative TG (DTG), and DSC curves were recorded continuously as functions of temperature.

Raw thermal data were processed in R (v4.3.0). Data were converted to long format (retaining all valid temperature points). To compare curves between populations, bootstrap resampling (2,000 iterations) was used to estimate the mean and 95% confidence intervals of TG and DTG at each temperature. For each biological replicate, key thermal features were extracted, including the minimum mass loss in TG, the maximum degradation rate in DTG, and the maximum endothermic/exothermic peak values in DSC, along with their corresponding temperatures. All statistical comparisons were performed at the level of biological replicates (*n* = 4 per group). Bootstrap-derived or curve-interpolated values were used exclusively for visualization and estimation of confidence intervals, not for hypothesis testing. Group differences in extracted features were tested using two-sample *t*-tests; when assumptions of normality or homogeneity of variances were violated (Shapiro–Wilk tests; variance-equality tests), Wilcoxon rank-sum tests were used. Significance was set at *P* < 0.05.

### Statistical analysis

Morphological traits quantified from SEM images included barbule length, hamuli number, and barbule density across all four feather regions. Variable distributions were checked prior to modeling. Categorical predictors were population, sex, and feather region. To avoid pseudoreplication caused by repeated measurements within individuals, we fitted linear mixed-effects models (LMMs) for continuous responses and generalized linear mixed-effects models (GLMMs) for count responses. Individual identity was included as a random intercept in all models to account for non-independence. Fixed effects included population, feather region, and sex as additive main effects (no interaction terms were included in the final models). For traits not measured across all regions (cilia and ventral teeth: tail and wing only; internode length and width: back and breast only), models were fitted using the corresponding data subset, and the feather-region factor was restricted to the observed levels.

For barbule length and density, model diagnostics indicated minor deviations from homoscedasticity and residual uniformity assumptions; however, the additive structure was retained to maintain model parsimony given limited sample size. For hamuli number, an initial Poisson GLMM exhibited significant overdispersion (*P* < 0.05), so we used a negative binomial GLMM (glmmTMB with nbinom2 family). For ventral teeth number, a negative binomial GLMM was used to account for mild overdispersion in count data. Final models were validated *via* DHARMa, confirming residual uniformity (Kolmogorov–Smirnov test *P* > 0.05) and homoscedasticity (*P* > 0.05). Fixed effects were evaluated using Type-III Wald *χ*^2^ tests. Cohen’s *d* values were calculated using model-derived residual variance (standardized mean differences based on LMM/GLMM estimates), and 95% confidence intervals were obtained by delta approximation. When significant main effects were detected, estimated marginal means (EMMs) were obtained using the *emmeans* package, and *post hoc* comparisons were performed with Tukey adjustment for multiple testing. These comparisons included pairwise main-effect contrasts only and, for traits measured across four regions (hamuli number, barbule length, and barbule density), pairwise contrasts among feather regions. All tests were two-tailed with a significance threshold of *p* < 0.05. All analyses were conducted in R, and figures were generated using the *ggplot2* package (v5.2.0; R Core Team, 2023).

## Results

### Variation in hamuli number, barbule length, and barbule density

Across both HA and LA ETSs, feather regions differed in barbule organization. Tail and wing feathers contained interlocking structures (hamuli, cilia, ventral teeth), whereas back and breast feathers showed nodal structures in their downy regions (Fig. 1). For the three traits in all four feather regions, hamuli number varied among feather regions but showed no effects of population, sex, or any interactions (Table 1). *Post hoc* comparisons indicated that hamuli number was similar between tail and wing feathers and between back and breast feathers; however, tail and wing feathers had more hamuli than back and breast feathers (Fig. 2). In contrast, barbule length and barbule density also showed strong feather-region effects, with no significant effects of population or sex (Table 1). *Post hoc* tests revealed strong feather region-specific patterns: barbules were the longest in tail feathers, followed by wing and back feathers, and the shortest in breast feathers, whereas barbule density exhibited the opposite reverse pattern (Fig. 2). Notably, no significant population- or sex-related differences were detected within feather regions for these traits (Table S1).

**Figure 1:**
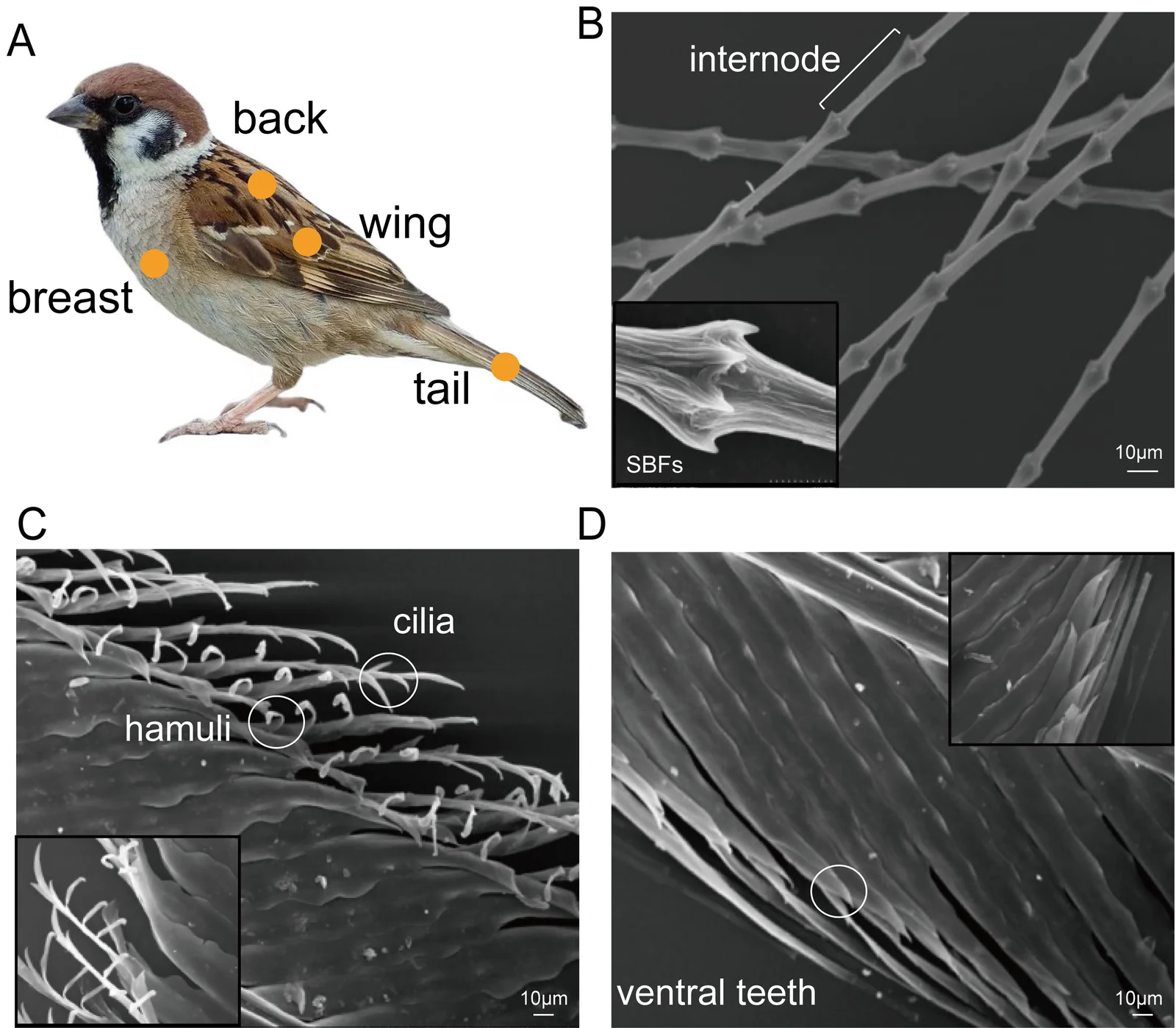
Feather microstructure across feather regions in the Eurasian tree sparrow (*Passer montanus*). (A) Schematic showing the four feather sampling regions: back, breast, wing (remiges), and tail (rectrices). (B) Scanning electron micrograph (SEM) of a back feather highlighting a representative internode; inset shows higher-magnification view of syncytial barbule fibers (SBFs). (C) SEM of a wing feather showing hooked barbules bearing hamuli and paired cilia (circled); inset shows a higher-magnification view of the interlocking structures. (D) SEM of a wing feather showing unhooked barbules with ventral teeth (circled); inset shows a higher-magnification view of ventral teeth. Scale bars: 10 µm.

**Table 1:** Effects of feather region, population, and sex on internode length, and internode width, estimated using linear mixed models.

**Trait**	**Contrast**	**Estimate/Ratio ± SE**	***df***	***z/t*** ** value**	***P*** ** value**	**Cohen’s d**	**95% CI**
Hamuli number	Back–Breast	0.95 ± 0.07	–	–0.70	0.897	0.11	(−0.25, 0.47)
	Back–Tail	0.75 ± 0.05	–	–4.10	**<0**.**001**	0.62	(0.41, 0.83)
	Back–Wing	0.66 ± 0.05	–	–6.04	**<0**.**001**	0.91	(0.68, 1.14)
	Breast–Tail	0.79 ± 0.05	–	–3.54	**0**.**002**	0.54	(0.32, 0.76)
	Breast–Wing	0.69 ± 0.05	–	–5.56	**<0**.**001**	0.84	(0.61, 1.07)
	Tail–Wing	0.87 ± 0.05	–	–2.16	0.135	0.33	(0.10, 0.56)
Barbule length	Back–Breast	0.13 ± 0.03	548	4.92	**<0**.**001**	0.39	(0.28, 0.50)
	Back–Tail	−0.84 ± 0.03	548	−31.43	**<0**.**001**	−2.46	(−2.66, −2.26)
	Back–Wing	−0.49 ± 0.03	548	−18.32	**<0**.**001**	−1.43	(−1.58, −1.28)
	Breast–Tail	−0.98 ± 0.03	548	−36.30	**<0**.**001**	−2.84	(−3.05, −2.63)
	Breast–Wing	−0.62 ± 0.03	548	−23.21	**<0**.**001**	−1.82	(−1.98, −1.66)
	Tail–Wing	0.35 ± 0.03	548	13.11	**<0**.**001**	1.03	(0.90, 1.16)
Barbule density	Back–Breast	−6.39 ± 0.54	549	−11.93	**<0**.**001**	−1.11	(−1.26, −0.96)
	Back–Tail	9.07 ± 0.54	549	16.94	**<0**.**001**	1.58	(1.41, 1.75)
	Back–Wing	1.46 ± 0.54	549	2.74	**0**.**006**	0.26	(0.12, 0.40)
	Breast–Tail	15.46 ± 0.54	549	28.7	**<0**.**001**	2.69	(2.50, 2.88)
	Breast–Wing	7.85 ± 0.54	549	14.54	**<0**.**001**	1.37	(1.21, 1.53)
	Tail–Wing	−7.61 ± 0.54	549	−14.09	**<0**.**001**	−1.33	(−1.49, −1.17)

**Notes.**

Significant effects (*P* < 0.05) are shown in bold.

**Figure 2:**
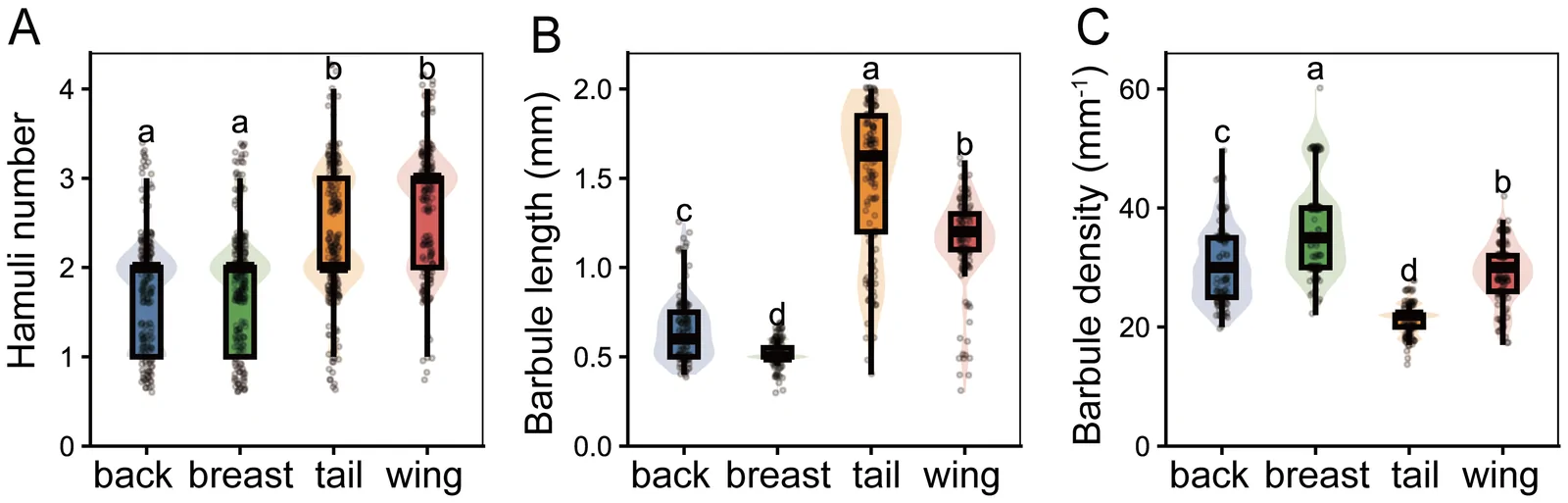
Comparisons of hamuli number, barbule length and density among feather regions. (A) Hamuli number across four feather regions (back, breast, tail, and wing). (B) Barbule length across feather regions. (C) Barbule density across feather regions. Data are shown as violin plots overlaid with boxplots and jittered individual observations. Violin plots depict kernel density distributions (scaled to equal maximum width); boxplots show medians and interquartile ranges, with whiskers indicating the data range (outliers omitted for clarity). Different letters above groups indicate significant pairwise differences among feather regions based on Tukey *post hoc* tests (*P* < 0.05). (A) Hamuli number across four feather regions (back, breast, tail, and wing). (B) Barbule length across feather regions. (C) Barbule density across feather regions. Data are shown as violin plots overlaid with boxplots and jittered individual observations. Violin plots depict kernel density distributions (scaled to equal maximum width); boxplots show medians and interquartile ranges, with whiskers indicating the data range (outliers omitted for clarity). Different letters above groups indicate significant pairwise differences among feather regions based on Tukey *post hoc* tests (*P* < 0.05).

### Variation in cilia number, ventral teeth number, and internode length and width

Across the two sampled vane regions (tail and wing), both cilia number and ventral teeth number did not vary with feather region or sex, but showed a significant population effect, with HA individuals having higher values than LA individuals (Figs. 3A, 3B; Table S2). The apparent bimodal patterns in the pooled distributions of these count traits may largely reflect the discrete, integer-valued nature of the measurements rather than clear separation by feather region or sex. For internode barbules in back and breast feathers, internode length showed a significant main effect of feather region but no significant effects of population or sex. Specifically, internodes were significantly longer in back feathers than in breast feathers (Fig. 3C; Table 2). Internode width also showed a significant main effect of feather region, with no significant effects of population or sex. Back-feather internodes were significantly wider than breast-feather internodes (Fig. 3D; Table 2).

**Figure 3:**
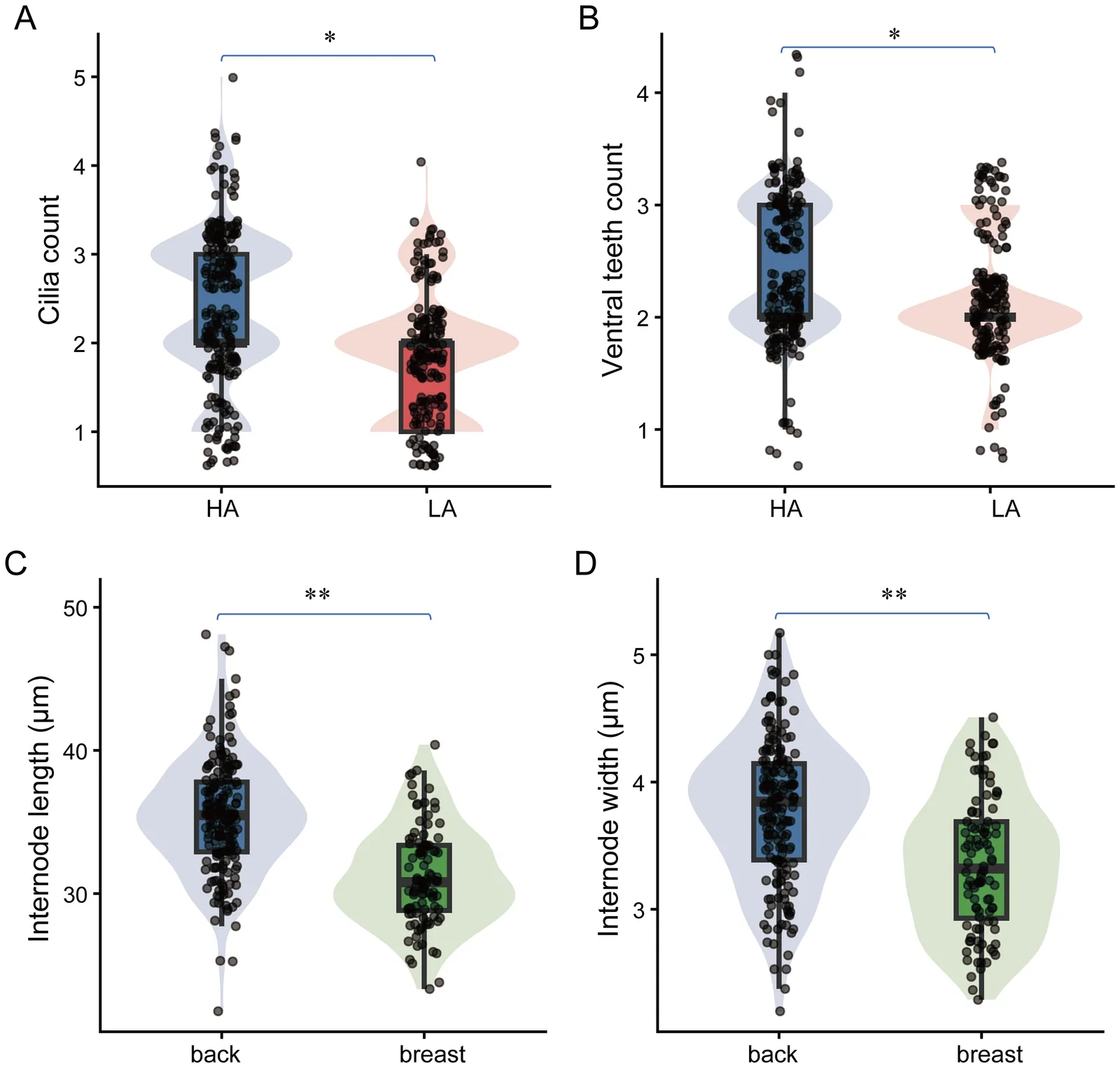
Population- and region -dependent variation in feather microstructural traits. (A) Cilia count per barbule in high-altitude (HA) and low-altitude (LA) populations. (B) Ventral teeth count per barbule in HA and LA populations. (C) Internode length (µm) in back and breast feathers. (D) Internode width (µm) in back and breast feathers. All panels are shown as violin plots overlaid with boxplots and jittered individual observations. Asterisks indicate significant differences for the indicated comparisons (*P < 0.05; **P < 0.01).

**Table 2:** Effects of feather region, population, and sex on hamuli number, barbule length, and barbule density, estimated using generalized linear mixed models (GLMMs) or linear mixed models (LMMs).

**Trait**	**Fixed effect**	**Estimate ± SE**	***df***	***t*** ** value**	***P*** ** value**	**Cohen’s d**		**95% CI**
Internode length	Population (LA *vs* HA)	−2.397 ± 1.347	4.78	−1.78	0.138	−0.36	(−0.62, −0.10)	
	Region (back *vs* breast)	−3.323 ± 0.467	273.71	−7.11	**<0**.**001**	−1.21	(−1.35, −1.07)	
	Sex (male *vs* female)	1.645 ± 1.347	4.78	1.22	0.279	0.25	(0.00, 0.50)	
Internode width	Population (LA *vs* HA)	−0.101 ± 0.242	4.87	−0.42	0.696	−0.04	(−0.18, 0.10)	
	Region (back *vs* breast)	−0.272 ± 0.069	275.92	−3.94	**<0**.**001**	−0.99	(−1.13, −0.85)	
	Sex (male *vs* female)	0.079 ± 0.242	4.87	0.33	0.758	0.03	(−0.11, 0.17)	

**Notes.**

Significant effects (*P* < 0.05) are shown in bold.

### Comparable thermal stability of breast feathers between HA and LA ETSs

Thermogravimetric analyses showed that HA and LA ETSs exhibited similar mass-loss trajectories across the full temperature range (Fig. 4A). Derivative thermogravimetry revealed two comparable major decomposition peaks in both populations (HA *vs.* LA: 342.83 °C *vs* 336.27 °C; 451.04 °C *vs* 450.91 °C; Fig. 4B). Differential scanning calorimetry likewise showed qualitatively similar thermal-decomposition profiles between HA and LA breast feathers (Fig. 4C; Table 3).

**Figure 4:**
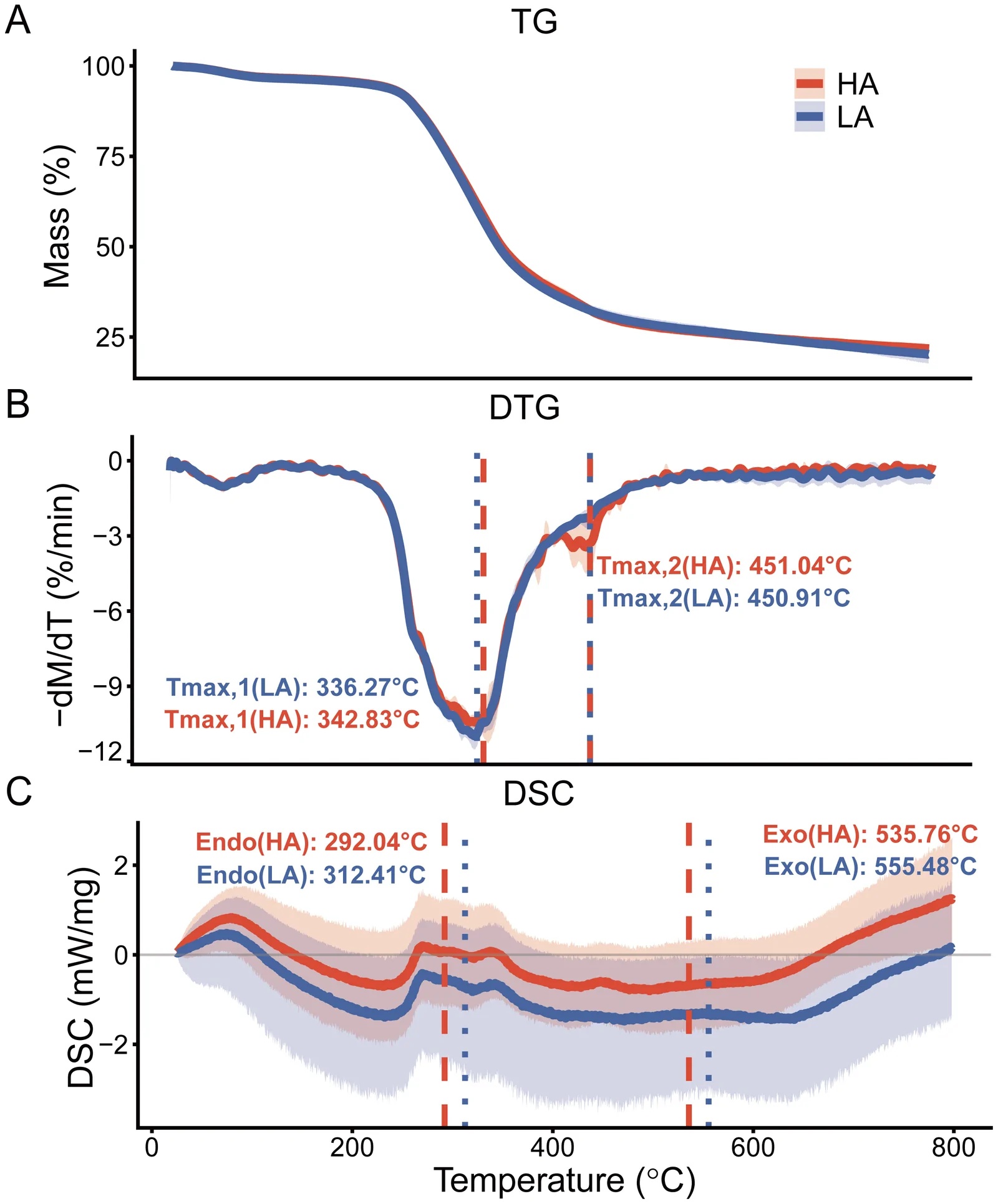
Thermal behavior of breast feathers from high- (HA) and low-altitude (LA) Eurasian tree sparrows. (A) Thermogravimetric (TG) curves showing percent mass remaining as temperature increases. (B) Derivative thermogravimetric (DTG) curves showing two main decomposition-rate peaks (*T*_max_): *T*_max,1_ = 342.83 °C (HA) *vs* 336.27 °C (LA) and *T*_max,2_ = 451.04 °C (HA) *vs* 450.91 °C (LA). (C) Differential scanning calorimetry (DSC) curves showing one endothermic transition (Endo): 292.04 °C (HA) *vs* 312.41 °C (LA), and one exothermic peak (Exo): 535.76 °C (HA) *vs* 555.48 °C (LA). Shaded bands denote 95% bootstrap confidence intervals.

## Discussion

### Feather-region differences dominate microstructural variation

Across both HA and LA ETS populations, feather microstructure differed more strongly among regions than between populations, supporting the long-standing view that tract-specific functional specialization is a primary axis of plumage variation (Lucas & Stettenheim, 1972; Videler, 2005). This pattern is consistent with developmental and evo-devo frameworks suggesting that feather tracts are organized through conserved integumentary patterning systems, in which regional differences emerge from spatially regulated developmental modules rather than fully independent morphological evolution (Chen et al., 2021; Dhouailly, 2023; Chuong et al., 2025). The clear separation we observed between wing/tail feathers *versus* back/breast feathers matches the expectation that aerodynamic tracts experience distinct mechanical demands compared with primarily protective/insulative body tracts (Broggi et al., 2011; Hendrickx-Rodriguez & Lentink, 2025). Similar region-by-region differentiation is also consistent with evidence that different performance requirements shape body feathers and flight feathers and often respond differently to environmental or developmental constraints (Broggi et al., 2011). Given the limited biological replication (*n* = 4 per population), these patterns should be interpreted as indicative case-level trends rather than definitive population-level inferences. Moreover, because Eurasian tree sparrows are relatively recent colonists of the Qinghai–Tibet Plateau (∼2,600 years; Qu et al., 2020), future comparative studies involving long-resident high-altitude taxa would be needed to test whether colonization history influences the tempo of flight-feather microstructural divergence.

Tail and wing feathers consistently exhibited the canonical vane “interlocking” toolkit—hooked barbules bearing hamuli and paired cilia, plus unhooked barbules with ventral teeth. This structural system underlies vane integrity, reversible attachment, and controlled permeability during aerodynamic loading (Lentink et al., 2007; Sullivan et al., 2016; Matloff et al., 2020). Such differentiation is consistent with functional and evolutionary frameworks, indicating that aerodynamic feathers are subject to stronger mechanical constraints, whereas body feathers may retain greater plasticity in traits related to insulation (Videler, 2005; Terrill & Shultz, 2023). Mechanistically, hooklets and associated barbule specializations act as a repeatable attachment network that helps maintain a continuous vane while allowing local separation and reattachment as feathers overlap and shift during tail and wing morphing (Lentink et al., 2007; Matloff et al., 2020). This is consistent with our finding that hamuli were much more abundant in tail and wing feathers than in back and breast feathers. In contrast, back and breast feathers largely lacked pronounced interlocking specializations but displayed nodal barbules with periodic nodes and spinous processes. Such structures are commonly associated with the downy/insulative component of body plumage, which primarily traps air and modulates porosity rather than maintaining a sealed aerodynamic surface (King & Farner, 1961; Wolf & Walsberg, 2000). In this context, weaker selection for tight vane cohesion in back and breast feathers provides a straightforward explanation for the reduced investment in interlocking structures relative to tail and wing feathers (Stettenheim, 2000). This tract-dependent organization fits classic descriptions of pennaceous feather architecture and the differing mechanical demands placed on aerodynamic *versus* primarily protective/insulative body plumage (Lucas & Stettenheim, 1972; Gill, 2007).

**Table 3:** Comparisons of peak temperature and corresponding thermal values between high- and low-altitude Eruasian tree sparrows (HA–LA), based on two-tailed Student’s t-tests.

**Factor**	**Contrast**	**Estimate/Ratio ± SEM**	***df***	***t*** ** value**	***P*** ** value**
DTG1 (250–350 °C)	HA–LA	6.56 ± 1.97	6	1.47	0.168
DTG2 (450–550 °C)	HA–LA	0.13 ± 0.58	6	0.22	0.833
DSC Endo (250–400 °C)	HA–LA	−20.37 ± 20.28	6	−1.00	0.337
DSC Exo (500–650 °C)	HA–LA	−19.72 ± 34.50	6	−0.57	0.579
DTG1 Value	HA–LA	0.46 ± 0.31	6	1.48	0.165
DTG2 Value	HA–LA	−1.10 ± 0.56	6	−1.96	0.088
DSC Endo Value	HA–LA	−0.64 ± 0.97	6	0.66	0.520
DSC Exo Value	HA–LA	−1.57 ± 1.98	6	0.79	0.445

Region-level contrasts in quantified traits further reinforce this functional partitioning. The higher hamuli number and longer barbules in tail and wing feathers provide a direct microstructural basis for stronger vane coherence and load sharing in aerodynamic tracts (Kovalev, Filippov & Gorb, 2014; Stettenheim, 2000; Sullivan et al., 2016). Meanwhile, the opposing gradients of barbule length (tail longest; breast shortest) and barbule density (breast highest; tail lowest) suggest a tract-dependent trade-off between producing a stiff, coherent vane and producing a surface with different permeability/loft properties (D’Alba et al., 2014; Videler, 2005). Collectively, these results caution against inferring “feather adaptation” from a single tract, as baseline microstructural architectures differ fundamentally across feather regions (Lingham-Soliar, 2019; Videler, 2005).

### Limited altitude- and sex-associated divergence suggests a strong constraint on vane-interlocking traits

High-altitude environments impose simultaneous challenges to thermoregulation (cold) and flight (reduced air density), creating *a priori* expectations of population divergence in plumage traits (Altshuler & Dudley, 2006; Scott, 2011; Kiat, Vortman & Sapir, 2019; Martin et al., 2023; Cheng et al., 2026). However, within our sampling effort, most vane-interlocking traits in tail and wing feathers showed limited divergence between populations and sexes. Cilia number showed a statistically detectable population effect, ventral teeth number showed a weaker population-associated signal, whereas sex and region effects were generally non-significant for both traits. This pattern is consistent with the possibility that structures involved in vane integrity and feather overlap/permeability during flight are subject to functional constraints and may therefore tolerate only limited divergence without compromising aerodynamic performance (Videler, 2005; Lentink et al., 2007; Matloff et al., 2020). Biomechanical and structural studies of flight feathers similarly indicate that vane formation relies on integrated and mechanically optimized interlocking systems that maintain a continuous but deformable aerodynamic surface (Zhang, Jiang & Wang, 2018; Chang et al., 2019; Matloff et al., 2020).

Barbule length and density also varied primarily among feather regions, with no consistent population- or sex-associated differences within individual feather regions. This further indicates that feather-tract identity, rather than altitude or sex, was the dominant axis of microstructural variation in our dataset. Our findings differ from those reported in great tits (*Parus major*), in which barbule density varied between environments, although those differences were influenced by individual age (Sándor et al., 2021; Sándor et al., 2022). Because age was not controlled in the present study, future work should examine whether age-related variation contributes to feather microstructure. Overall, altitude-associated differences were detectable only in specific interlocking traits and were modest relative to the strong region-specific patterns. It is therefore possible that altitude-related effects are subtle, partly masked by individual variation, or expressed in unmeasured traits (*e.g.*, feather thickness, barb diameter, rachis mechanics, whole-plumage depth, or feather mass). In contrast, variation in body-feather internodal traits was spatially restricted and did not involve the core vane-interlocking systems, reinforcing a general distinction between relatively conserved aerodynamic structures and more variable insulative architectures. This pattern is consistent with broader frameworks emphasizing that selection and plasticity may act on multiple organizational levels, and that not all levels respond detectably within a given dataset (Monaghan, 2012; Pigliucci, 2010).

### Altitude- and sex-dependent signals emerge in nodal features of body feathers

In contrast to the relatively conserved interlocking traits of tail and wing feathers, internodal traits of body feathers showed clear regional differentiation but limited evidence of population- or sex-associated divergence. Breast internodes were longer and wider than back internodes, indicating strong regional structuring of body feather architecture. However, population and sex effects were not statistically supported in the additive models. Although breast internode length tended to be greater in high-altitude birds and in females, these patterns should be interpreted cautiously because they were not significant in the final model structure. Variation in internode geometry may influence plumage loft, barb spacing, local compressibility, and air-trapping capacity, all of which can contribute to insulation under cold conditions. Such a possibility is consistent with the expectation that high-altitude environments may increase the functional importance of body plumage, particularly in ventral/breast tracts closely tied to heat balance, whereas aerodynamic tracts may remain more constrained (Scott, 2011; Pap et al., 2015; Pap et al., 2020). Comparative studies across elevational gradients also suggest that insulative feather traits, often reflected by greater investment in the plumulaceous or downy component, can increase in colder, higher-elevation environments, although responses vary among taxa and ecological contexts (Barve et al., 2021; Sándor et al., 2022). Reports of weak or absent altitudinal differentiation in contour-feather structure in certain systems (*e.g.*, torrent ducks) further underscore that such responses are lineage- and ecology-dependent (Sándor et al., 2022).

The apparent tendency for longer breast internodes in females was not statistically supported and should therefore not be interpreted as evidence of a sex-specific effect (Badyaev et al., 2001; Badyaev & Hill, 2003; Pigliucci, 2010). More generally, the functional consequences of internode variation are unlikely to be monotonic or attributable to a single microstructural trait. Insulation emerges from integrated plumage properties (loft, porosity, overlap, compressibility), and microstructural changes may reflect trade-offs among warmth, durability, and water shedding (Wolf & Walsberg, 2000). We also acknowledge that observed microstructural patterns may arise from adaptive processes, neutral genetic drift, developmental constraints, or environmentally induced plasticity, and our dataset does not allow these alternatives to be formally distinguished. Direct thermal conductance or air-permeability measurements would be valuable steps for linking internode geometry to functional performance.

### Comparable thermal decomposition profiles imply conserved thermal stability across altitude

Our TGA/DTG/DSC profiles of breast feathers were qualitatively similar between HA and LA populations, suggesting broadly conserved thermal decomposition behavior and no detectable population-level divergence in bulk feather thermal stability (Raydan et al., 2024; Takahashi et al., 2004). This interpretation is consistent with thermal characterizations of feather β-keratins showing reproducible multistage decomposition patterns, and with the general biochemical commonality of keratinous materials across conspecifics (Chilakamarry et al., 2021; Staroszczyk et al., 2025). It is also consistent with the broader view that avian β-keratins are structurally conserved despite diversification of their gene families, with core motifs and assembly mechanisms maintained under strong functional constraints (Greenwold et al., 2014; Ng et al., 2014; Wu et al., 2015a; Wu et al., 2015b; Parry, 2026). Consequently, the population differences we detected in body-feather internode traits are more parsimoniously explained by architectural (microstructural) variation than by changes in the underlying keratin matrix detectable *via* bulk thermal analysis (Banasaz & Ferraro, 2024; Hendrickx-Rodriguez & Lentink, 2025). Therefore, any population-level microstructural variations observed are unlikely to substantially influence the feather’s thermoregulatory function by altering the material’s inherent thermal properties, suggesting that any adaptive value lies in morphological rather than material changes.

## Limitations

We acknowledge several limitations. First, SEM-based microstructural analyses were conducted on a limited number of individuals, with only two males and two females per population, which restricts statistical power and limits the robustness of inferences regarding population- and sex-related differences. Models with higher-order interactions can be sensitive to sample size. Second, altitude covaries with other environmental factors (*e.g.*, humidity, wind exposure, diet, and urbanization), any of which could contribute to population differences. Third, our measurements capture only a subset of feather architecture; additional traits (*e.g.*, barb diameter, vane thickness, rachis properties, three-dimensional porosity, and whole-feather bending/torsion mechanics) and direct functional assays (*e.g.*, insulation, air permeability, and wear resistance) are needed to link microstructure to performance. Fourth, thermal analysis provides only a coarse proxy for composition. Complementary chemical assays (*e.g.*, amino-acid profiling, elemental analysis, and targeted measures of disulfide content or crosslinking) would help test for subtle biochemical differences that may not be resolved by similar TGA/DTG/DSC profiles. We therefore emphasize that our results should be interpreted as an exploratory case study of the sampled individuals rather than a definitive population-level characterization of ETSs across altitudes.

## Conclusions

By comparing four feather regions (back, breast, tail, and wing) and both sexes across HA and LA populations of ETSs, this study provides an exploratory assessment of feather microstructural variation across an altitudinal contrast using a combined morphological and materials-science framework. Our findings provide preliminary evidence that feather microstructure is strongly region-specific, underscoring the functional modularity of plumage. Across the altitudinal contrast, tail and wing traits central to vane interlocking (hamuli number, cilia number, and ventral teeth number), as well as barbule length and density, appeared largely invariant within our sampling, consistent with strong aerodynamic constraint. In contrast, altitude- and sex-dependent divergence was limited and localized to body-feather nodal architecture: HA females had longer breast internodes relative to LA females, and breast internodes were narrower than back internodes in LA birds but not in HA birds. Breast-feather thermal decomposition profiles were comparable between populations, suggesting conserved keratin thermal stability and implying that any adaptive differentiation is more likely attributable to microstructural architecture than to major shifts in keratin composition. Whether similar patterns occur across broader populations, seasons, age classes, or other HA taxa remains to be determined.

##  Supplemental Information

10.7717/peerj.21803/supp-1 Supplemental Information 1 Detailed pairwise comparisons and regional microstructure analyses

## Additional Information and Declarations

### Data Availability

The following information was supplied regarding data availability: The data is available at Zenodo: - Liu, F., Zhang, T., Liu, X., Zhang, Q., Zhang, T., Li, J., Zhao, M., Kou, G., Wang, L., Wang, Y., & Li, D. (2026). An exploratory comparison of region-specific feather microstructure in two Eurasian tree sparrow populations across altitudes [Data set]. Zenodo. https://doi.org/10.5281/zenodo.18506066.
