# Supplementary material for: An exploratory comparison of region-specific feather microstructure in two Eurasian tree sparrow populations across altitudes
Source: PeerJ. 2026 Oct 22;14:e21803. doi: 10.7717/peerj.21803 (PMC13695958; doi:10.7717/peerj.21803)
Supplement: Supplementary file 1 [file peerj-14-21803-s001.docx]

**Table S1 Effects of population and sex on hamuli number, barbule length, and barbule density**

| **Trait** | **Fixed effect** | **Estimate ± SE** | ***df*** | ***z / t* value** | ***P* value** | **Cohen’s d** | **95% CI** |
| --- | --- | --- | --- | --- | --- | --- | --- |
| Hamuli number | Population (LA vs HA) | 1.02 ± 0.057 | – | 0.338 | 0.735 | 0.05 | (-0.06, 0.16) |
|  | Sex (male vs female) | 1.02 ± 0.055 | – | 0.371 | 0.710 | 0.06 | (-0.05, 0.17) |
| Barbule length | Population (LA vs HA) | 0.008 ± 0.077 | 5.02 | 0.109 | 0.918 | 0.01 | (-0.14, 0.16) |
|  | Sex (male vs female) | 0.091 ± 0.077 | 5.02 | 1.192 | 0.287 | 0.12 | (-0.03, 0.27) |
| Barbule density | Population (LA vs HA) | 2.41 ± 2.60 | 4.99 | 0.928 | 0.396 | 0.09 | (-0.12, 0.30) |
|  | Sex (male vs female) | -2.04 ± 2.60 | 4.99 | -0.785 | 0.468 | -0.08 | (-0.29, 0.13) |

**Table S2 Effects of population, feather region, and sex on cilia number and ventral teeth number in Eurasian tree sparrows**

| **Trait** | **Fixed effect** | **Estimate ± SE** | ***df*** | ***z* value** | ***P* value** | **Cohen’s d** | **95% CI** |
| --- | --- | --- | --- | --- | --- | --- | --- |
| Cilia count | Population (LA vs HA) | −0.238 ± 0.093 | – | −2.57 | **0.010** | -0.49 | (-0.67, -0.31) |
|  | Region (tail vs wing) | −0.052 ± 0.069 | – | −0.75 | 0.452 | -0.10 | (-0.24, 0.04) |
|  | Sex (male vs female) | −0.160 ± 0.091 | – | −1.76 | 0.079 | -0.33 | (-0.51, -0.15) |
| Ventral teeth count | Population (LA vs HA) | −0.132 ± 0.067 | – | −1.98 | **0.048** | -0.39 | (-0.53, -0.25) |
|  | Region (tail vs wing) | 0.095 ± 0.066 | – | 1.44 | 0.149 | 0.27 | (0.14, 0.40) |
|  | Sex (male vs female) | −0.025 ± 0.065 | – | −0.38 | 0.707 | -0.07 | (-0.20, 0.06) |

Significant effects (*P* < 0.05) are shown in bold.
